# Not Only Diagnostic Yield: Whole-Exome Sequencing in Infantile Cardiomyopathies Impacts on Clinical and Family Management

**DOI:** 10.3390/jcdd9010002

**Published:** 2021-12-21

**Authors:** Laura Pezzoli, Lidia Pezzani, Ezio Bonanomi, Chiara Marrone, Agnese Scatigno, Anna Cereda, Maria Francesca Bedeschi, Angelo Selicorni, Serena Gasperini, Paolo Bini, Silvia Maitz, Carla Maccioni, Cristina Pedron, Lorenzo Colombo, Daniela Marchetti, Matteo Bellini, Anna Rita Lincesso, Loredana Perego, Monica Pingue, Nunzia Della Malva, Giovanna Mangili, Paolo Ferrazzi, Maria Iascone

**Affiliations:** 1 Laboratorio di Genetica Medica, ASST Papa Giovanni XXIII, 24127 Bergamo, Italy; lpezzoli@asst-pg23.it (L.P.); lidiapezzani@yahoo.it (L.P.); dmarchetti@asst-pg23.it (D.M.); m.bellini@asst-pg23.it (M.B.); arlincesso@asst-pg23.it (A.R.L.); lperego@asst-pg23.it (L.P.); monicapingue@hotmail.it (M.P.); ndellamalva@asst-pg23.it (N.D.M.); 2 Pediatria ad Alta Intensità di Cura, Fondazione IRCCS Ca’ Granda Ospedale Maggiore Policlinico, 20122 Milano, Italy; 3 Terapia Intensiva Pediatrica, ASST Papa Giovanni XXIII, 24127 Bergamo, Italy; ebonanomi@asst-pg23.it; 4 Cardiologia Pediatrica, Fondazione G. Monasterio, 54100 Massa, Italy; marrone@ftgm.it; 5 Pediatria, ASST Papa Giovanni XXIII, 24127 Bergamo, Italy; ascatigno@asst-pg23.it (A.S.); acereda@asst-pg23.it (A.C.); 6 Genetica Medica, Fondazione IRCCS Ca’ Granda Ospedale Maggiore Policlinico, 20122 Milano, Italy; mariafrancesca.bedeschi@policlinico.mi.it; 7 Pediatria, ASST Lariana, 22100 Como, Italy; angelo.selicorni61@gmail.com; 8 Malattie Metaboliche Rare, Dipartimento di Pediatria, Fondazione MBBM, ASST, 20900 Monza, Italy; serena.gasperini69@gmail.com; 9 Terapia Intensiva Neonatale, ASST Lariana, 22100 Como, Italy; paolo.bini@asst-lariana.it; 10 Ambulatorio di Genetica Pediatrica, Clinica Pediatrica, Fondazione MBBM, Ospedale S. Gerardo, 20900 Monza, Italy; maitz.silvia@gmail.com; 11 Terapia Intensiva Neonatale, Ospedale A. Manzoni, ASST, 23900 Lecco, Italy; c.maccioni@asst-lecco.it; 12 Cardiologia, Ospedale di Bolzano, Azienda Sanitaria dell’Alto Adige, 39100 Bolzano, Italy; CRISTINAMARIA.PEDRON@sabes.it; 13 NICU Fondazione IRCCS Ca’ Granda Ospedale Maggiore Policlinico, 20122 Milano, Italy; lorenzo.colombo@policlinico.mi.it; 14 Patologia Neonatale, ASST Papa Giovanni XXIII, 24127 Bergamo, Italy; gmangili@asst-pg23.it; 15 Centro Cardiomiopatia Ipertrofica, Policlinico di Monza, 20900 Monza, Italy; paolo.ferrazzi@policlinicodimonza.it

**Keywords:** whole-exome sequencing, infantile cardiomyopathy, urgent WES, clinical utility

## Abstract

Whole-exome sequencing (WES) is a powerful and comprehensive tool for the genetic diagnosis of rare diseases, but few reports describe its timely application and clinical impact on infantile cardiomyopathies (CM). We conducted a retrospective analysis of patients with infantile CMs who had trio (proband and parents)-WES to determine whether results contributed to clinical management in urgent and non-urgent settings. Twenty-nine out of 42 enrolled patients (69.0%) received a definitive molecular diagnosis. The mean time-to-diagnosis was 9.7 days in urgent settings, and 17 out of 24 patients (70.8%) obtained an etiological classification. In non-urgent settings, the mean time-to-diagnosis was 225 days, and 12 out of 18 patients (66.7%) had a molecular diagnosis. In 37 out of 42 patients (88.1%), the genetic findings contributed to clinical management, including heart transplantation, palliative care, or medical treatment, independent of the patient’s critical condition. All 29 patients and families with a definitive diagnosis received specific counseling about recurrence risk, and in seven (24.1%) cases, the result facilitated diagnosis in parents or siblings. In conclusion, genetic diagnosis significantly contributes to patients’ clinical and family management, and trio-WES should be performed promptly to be an essential part of care in infantile cardiomyopathy, maximizing its clinical utility.

## 1. Introduction

From their implementation in clinical practice until recently, genetic tests have always had a very long turnaround time due to their intrinsic technical characteristics and the common need for a selection of analyzed genes based on the proband’s clinical features. For these reasons, patients’ clinical management has rarely relied on the genetic testing result, and the latter has always been considered part of the diagnostic workflow, mainly for counseling purposes and for addressing a specific follow-up.

The introduction of next-generation sequencing in the genetic landscape has significantly reduced the turnaround time and improved the comprehensiveness of testing. In recent years, there has been an increasing number of studies that have shown the feasibility of exome sequencing (WES) or genome sequencing (WGS) performed urgently (turnaround time of approximately 12–23 days for WES and 16 h–7 days for WGS) with a diagnostic yield ranging from 22% to 57%, based on the patient’s phenotype and characteristics of the study cohort. These studies have demonstrated the impact of the rapid molecular diagnosis on clinical decision-making for critically ill children suspected of having a genetic disease [1,2,3,4,5].

Pediatric cardiomyopathies (CM) may represent a suitable population that could benefit from extensive and urgent genetic testing due to the considerable clinical and genetic heterogeneity and the severity of the underlying disease. Indeed, in children, CM ranges from isolated myocardial diseases to systemic conditions, with up to 50% of cases estimated to be affected by a plethora of rare metabolic, neuromuscular, mitochondrial, or syndromic disorders with time-demanding clinical diagnoses [6,7,8,9]. The earlier the symptoms appear, the more likely at the base there is a systemic condition with a poor prognosis [9], with more than 40% progressing to death or heart transplant (HTX) within two years of diagnosis [8,10].

Unraveling the molecular basis of CM is essential for prognosis evaluation and choice of treatment options. A comprehensive genetic assessment can be beneficial because features of the syndromes may be overlooked at the initial presentation, particularly in infants or in critically ill children [6]. In addition, children can sometimes be affected by a rapidly progressive form of CM, especially those with onset in the first year of life, so early diagnosis can be essential to promptly address medical management and for family genetic counseling [11]. Traditionally, the genetic testing of CM infants is directed at a single gene after extensive clinical investigation or focused on commercial gene panels based on common causes of adult cardiomyopathies [12]. By this approach, the etiological diagnosis of rare conditions leading to infantile CM is often delayed by the difficulty of suspecting the disease exclusively on clinical grounds, reducing the potential clinical impact of the genetic diagnosis, especially in those rare cases for which a specific therapy exists.

In the present study, we describe the results of the proband and parents (trio) WES analyses in infants affected by CM in urgent settings for patients admitted to the intensive care unit (ICU) due to acute presentation of heart failure, and non-urgent settings for patients with a more stable condition. The aim is to evaluate the clinical utility of timely trio-WES and its added value in achieving a definitive diagnosis and the consequent impact on clinical and family management.

## 2. Materials and Methods

### 2.1. Patients

Patients were recruited from 10 clinical centers and trio-WES analysis was performed in a single genetic laboratory for all patients. This multicenter study complied with the Declaration of Helsinki and was approved by the Ethics Committee of ASST Papa Giovanni XXIII of Bergamo as part of the RARE project (Rapid Analysis for Rapid Care). Informed written consent was obtained from the parents of the subjects during pre-test genetic counseling.

Echocardiographic diagnostic criteria defined the hypertrophic cardiomyopathy (HCM) phenotype based on wall thickness z scores computed as the number of SDs from the mean value relative to body surface area in a normal population, according to the current guideline definitions [6,13,14,15].

The dilated cardiomyopathy (DCM) phenotype was defined by echocardiography as an LV fractional shortening (LVFS) 2 standard deviations (SD) for age and an LV end-diastolic dimension (LVEDD) or volume 2 SD for body surface area, according to The Pediatric Cardiomyopathy Registry (PCMR) definitions [6,13].

Patients with decompensated heart failure (HF) symptoms and low cardiac output, or intractable arrhythmia refractory to medical therapy were admitted to ICU, considered for mechanical cardiac support or heart transplant (HF stage D), according to the current guidelines [16,17], and sent for urgent WES analysis.

Forty-two consecutive patients ≤ 12 months of age affected by CM underwent trio-WES analysis over eight years (2012–2020). No patients had previously performed other genetic tests. Before WES analysis, a complete clinical investigation has been performed in all patients, including an evaluation by a clinical geneticist. Three-generation pedigree, comprehensive patient and family history, developmental milestones, and physical examination data, together with biochemical and screening metabolic tests results, were collected. Each case was classified as isolated or complex (syndromic, mitochondrial, or inborn error of metabolism) CM and on the basis of the CM type. Patients with congenital structural heart diseases were excluded from this study.

### 2.2. Genetic Analyses

Genomic DNAs of the patients and their parents were extracted from peripheral blood using commercially available DNA extraction kits (Qiagen, Hilden, Germany). Exons and splice sites regions of the genome were enriched by the Agilent SureSelect Clinical Research Exome v1.0 (from year 2012 to 2016) and v2.0 (from year 2017 to 2020) enrichment kit (Agilent, Santa Clara, CA, USA), and 150 bp paired-end sequencing was performed on the NextSeq 500 platform (Illumina, San Diego, CA, USA). Sequence reads were mapped to the reference human genome assembly (February 2009, GRCh37/hg19) and analyzed by the BWA enrichment version 2.0 pipeline. The variant call file (vcf), including single nucleotide variants and indels, was annotated querying variants databases, including the Genome Aggregation Database (http://gnomad.broadinstitute.org/ (accessed on 1 October 2021)), ClinVar (https://www.ncbi.nlm.nih.gov/clinvar/ (accessed on 1 October 2021)), and Human Gene Mutation Database Professional (HGMD, Release 2020.2). We applied a sequential filtering strategy to prioritize the variants. We retained for further investigation only variants consistent with the patient’s phenotype, compatible with the inheritance model, and with a frequency in the general population consistent with the prevalence and incidence of the disease. Moreover, the candidate variants should have a pathogenic mechanism corresponding to the expected one for the disease.

All remaining negative probands were further analyzed for a selected panel of genes associated with cardiomyopathies (Cardiomyopathy PanelApp: https://panelapp.genomicsengland.co.uk/panels/749/, (accessed on 1 October 2021) green list).

Variants were classified based on the American College of Medical Genetics and Genomics (ACMG) guidelines [18,19]. Visual inspection was performed with Alamut Visual Software (http://www.interactive-biosoftware.com/alamut-visual/ (accessed on 1 October 2021) (version 1.4) and Integrative Genomic Viewer (version 2.8.0) [20].

Two pipelines were used to identify the CNVs based on ExomeDepth [21] and one created in-house. The in-house pipeline identifies CNVs analyzing the SAM files, particularly the MAPQ field, which reports the mapping quality score, and the TLEN field, which indicates the distance between the mapped end and the mapped start of the read. For detecting CNVs, we consider the reads with a mapping quality of 60 and a length between 2500 and 350,000 in absolute value. A prioritization is then made based on the number of reads called in the same region with very similar coordinates (±5%). All the CNVs detected by both pipelines were annotated by matching every call with the genes involved and related diseases and classified according to ACMG and ClinGen guidelines [22].

Variants of unknown significance (VUS) were not considered for the purposes of this work and by laboratory policy they are not listed in the final report unless they are strictly related to the patient’s phenotype and potentially causing disease or worthy of further investigation.

Following the European Society of Human Genetics (ESHG) recommendations, the analysis has been restricted to the original health problem, avoiding the opportunistic screening for variants unrelated to the phenotype of the proband (Incidental findings, IFs) [23].

The laboratory policy concerning VUS and IFs is detailed in the information sheet provided and explained to the families during the pre-test counseling.

Patients carrying pathogenic and likely pathogenic variants in a gene that the clinical team who ordered the test and cared for the child agreed it was the cause of the illness were considered to have received a genetic diagnosis. Pathogenic and likely pathogenic variants were confirmed in the proband and parents by Sanger sequencing using a second independent DNA sample. Negative cases were further analyzed by a second independent bioinformatics pipeline [24] with alignment towards the whole genome and variant calling not restricted to the exome, lower quality thresholds for variant calling, and no threshold for the variant allele frequency.

### 2.3. Turnaround Time and Clinical Utility Evaluation

**Figure 1:**
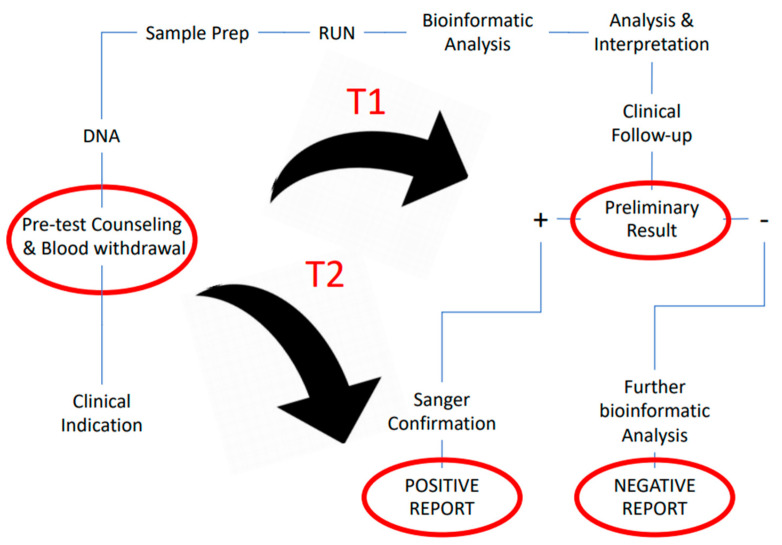
Measurement of turnaround time (TAT) of WES analysis in urgent and non-urgent settings. T1: time to preliminary result, T2: time to definitive report.

In the urgent setting, we measured time from the arrival of blood samples in the laboratory to the communication of preliminary results (T1). At this time, we also assessed the clinical utility of trio-WES results. Definitive report (T2) was provided after confirmation of the identified variant by Sanger sequencing for positive cases and after analysis with the second pipeline for negative cases. In the non-urgent setting, we registered solely T2 time (Figure 1).

The clinical utility of trio-WES was self-reported by the physician at the time of communication of the WES results (T1), which has been taken into account together with the patient’s clinical condition and the results of biochemical and instrumental tests for the final medical decision. The change in clinical management was picked up by the chart review and in agreement with the physician who ordered the test and cared for the child. The data were collected and recorded by clinical geneticists who are part of the clinical team.

## 3. Results

A total of 42 consecutive pediatric patients from 0 to 12 months (mean age 4.0 months, median 3.0 months) affected by CM underwent trio-WES analysis from 2012 to 2020. Twenty-six were male (61.9%) and 16 (38.1%) were female. Only five (11.9%) had a first or second-degree relative affected by CM based on family history. In particular, in four cases, the proband had an affected sibling, while in one, the affected relative was the mother. Prenatal detection of CM was reported in nine patients (21.4%).

In our population, the diagnosis of HCM (71.4%) was much more common than DCM (28.6%). CMs clinically classified as isolated were only 12 (28.6%, four HCM and eight DCM), while the majority of the patients (30; 71.4%) showed a complex phenotype, of which 19 (63.3%) were suspected to have a genetic syndrome and 11 (36.7%) an inborn error of metabolism (Table 1).

Twenty-four patients (57.1%) were admitted to ICU at the time of evaluations due to their critical clinical conditions characterized by acute decompensated HF symptoms and low cardiac output. The other 18 patients (42.9%) were in a stable clinical condition.

### 3.1. Overall Results

**Figure 2:**
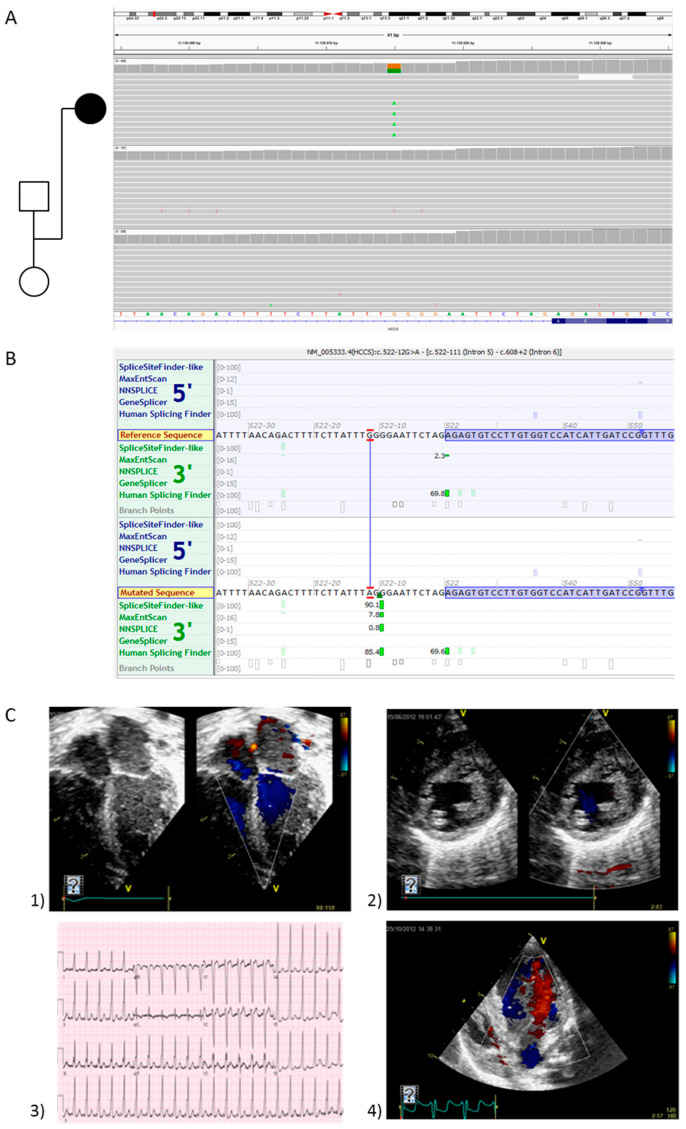
Clinical presentation and genetic findings of the patient with *HCCS* variant. (**A**) Trio-WES identified a *de novo* variant (NM_ 005333.4:c.522-12G>A) in *HCCS* gene (OMIM * 300056), predicted to create a new splice-site 12 bases upstream the canonical acceptor site of exon 6 (**B**). (**C**) Two-dimensional echocardiogram of patient #14 at birth (**1**) and 1 month later (**2**), showing mild left ventricular hypertrophy and dilation with apical non-compaction and mild mitral valve insufficiency, with normal right ventricle function and volumetry. Electrocardiogram (**3**) and echocardiogram (**4**) at 6 months of age, showing severe left ventricular dysfunction with a “foamy” appearance of the myocardium.

The diagnostic yield of trio-WES was 69%. The most frequent diagnosis was metabolic or mitochondrial disorder (10 out of the 29 positive ones; 34.5%), followed by sarcomeric CM (nine; 31%) and Rasopathy (eight; 27.6%). The remaining two patients (6.9%) were affected by ultra-rare syndromes caused by a truncating variant in NONO gene (OMIM #300967) and a splicing variant in HCCS gene (OMIM #309801) (Figure 2).

The list of pathogenic and likely pathogenic identified variants is reported in Table 2.

Of note, WES analysis also allowed to reclassify two apparently isolated CM that turned out to be complex ones (one Noonan syndrome, OMIM #163950, and one Pompe disease, OMIM #232300), and one suspected syndromic form that was actually a lethal cardiac glycogen storage disease (OMIM #261740), caused by a de novo variant in PRKAG2 gene.

**Figure 3:**
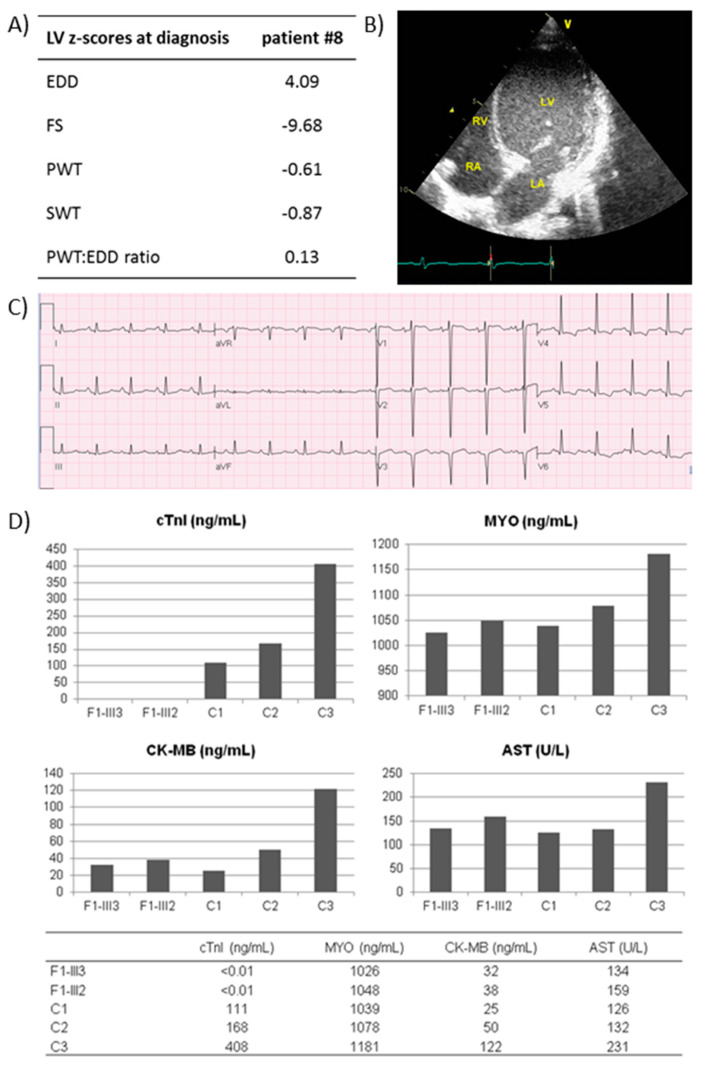
Clinical presentation and cTnI tissue levels of patients with homozygous *TNNI3* (OMIM *191044) variants. (**A**) Clinical data at admission of patient #8. (**B**) Two-dimensional echocardiogram of patient #8, showing a dilated left ventricle and left atrium, with rightward shift of the interatrial and interventricular septum and thin left free-walls. (**C**) Electrocardiogram of patient #8, showing low-voltage QRS complexes in inferior and lateral leads, no R wave progression in leads V1–V3 and T-wave inversion in leads V4–V6. (**D**) Histograms showing the tissue level of cTnI, myoglobin (MYO), muscular isoforms of creatine kinase (CK-MB) and aspartate aminotransferase (AST) in myocardial specimens from explanted frozen left ventricle of patient #41, her affected sister and three negative controls. F1-III3, patient #41; F1-III2, affected sister of patient #41; C1, age-matched hypertrophic cardiomyopathy patient; C2, age-matched idiopathic dilated cardiomyopathy patient; C3, adult ischemic cardiomyopathy patient. Abbreviations: LV, left ventricle; LA, left atrium; RV, right ventricle; RA, right atrium; EDD, end-diastolic dimension; FS, fractional shortening; PWT, posterior wall thickness; SWT, septal wall thickness.

In general, the outcome of the testing was associated with the presence of extracardiac features but not with a positive family history of cardiomyopathy. Indeed, de novo events have been responsible for about half of the cases (13; 44.8%) of diagnosed CM, with a substantial prevalence of syndromic CM. There were ten autosomal recessive forms of CM, of which seven inborn errors of metabolism or mitochondrial disorders and three isolated CMs due to homozygous variants led to the absence of the TNNI3 gene product (OMIM *191044) (Figure 3). There were two X-linked forms (6.9%; one syndromic and one metabolic) while the remaining four cases were isolated CM due to variants in the sarcomeric genes inherited from one (three cases) or both (one case) apparently healthy parent (s) with negative family history, for which a cardiological evaluation and follow-up has been arranged.

WES results facilitated a diagnosis in parents or siblings in seven cases: the four autosomal dominant sarcomeric CMs, two unrelated cases of recurrence of TNNI3 homozygous variant (isolated CM), and an inborn error of metabolism. All the families of the 29 diagnosed patients received specific counseling about recurrence risk, and three families underwent prenatal testing for subsequent pregnancies. Moreover, a specific and multidisciplinary follow-up was set up for all the probands.

**Figure 4:**
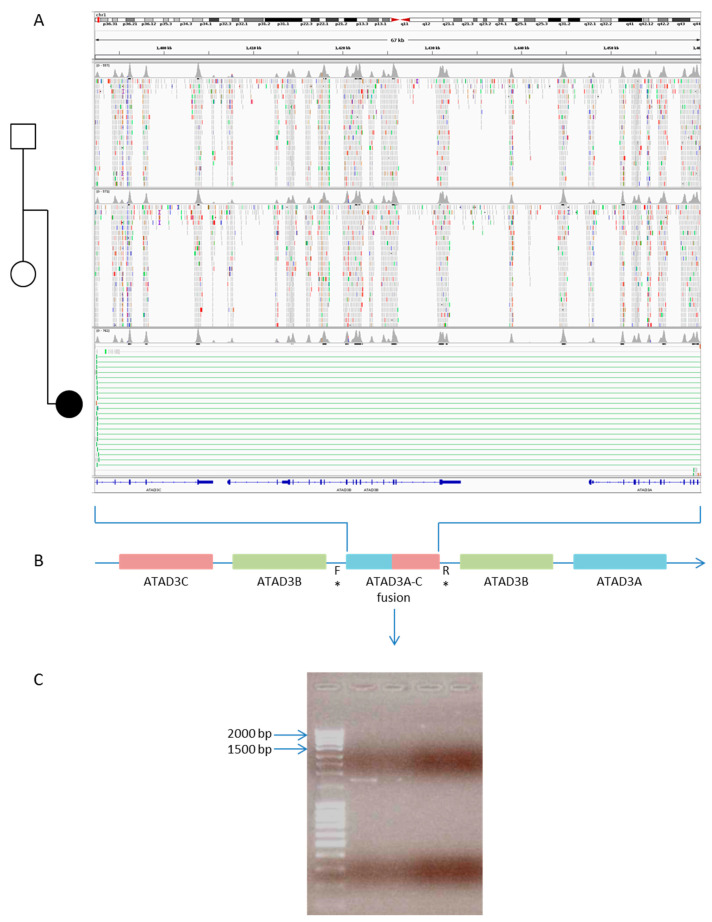
De novo duplication of the *ATAD3* gene cluster (OMIM #618815) identified in a female newborn with left ventricle hypertrophy and clinical suspicion of mitochondrial disease. (**A**) In a newborn female presenting with brain anomalies, left ventricular hypertrophy and corneal opacity, leading to the clinical suspicion of mitochondrial disease, we identified a previously reported de novo duplication in the *ATAD3* gene cluster (chr1:1392270_1460317dup), which creates an *ATAD3A-C* fusion gene (**B**). The presence of the duplication has been confirmed by PCR (**C**) using the same primers reported by Gunning et al. [25] for case 4 (* F: chr1 (GRCh37):1459103-1459123 and * R: chr1 (GRCh37):1392608-1392630).

Of note, the trio-WES approach allowed the detection of molecular defects causing ultra-rare or recently described conditions in three cases. We identified two syndromic CM, caused by variants in the NONO and HCCS genes and one due to the de novo duplication of the ATAD3 gene cluster (OMIM #618815) [25,26], mimicking a fatal mitochondrial heart failure (Figure 4). For none of these three conditions would the molecular diagnosis have been achieved using a traditional testing strategy, including serial clinical, biochemical, and instrumental evaluations, targeted genetic testing such as single gene analysis, or commercial genetic panels.

No incidental findings were identified in the study cohort.

### 3.2. Results in Urgent Setting

Among the 24 ICU critical patients, only five (20.8%) were isolated CMs, while 19 (79.2%) were complex forms. In particular, nine (eight HCM, one DCM) had a syndromic phenotype, while the other 10 (nine HCM, one DCM) were suspected metabolic disorders.

In these patients, a preliminary report has been provided to the clinicians on average in 9.7 days (T1, range 5–18 days; median 9.5 days), while the final report has been available averaged in 48 days (T2, range 18–120 days), with a total detection rate of 70.8% (17 out of 24). All the preliminary results provided at T1 were confirmed at T2 and no additional positive cases were identified.

The most frequent diagnosis in critical patients was a metabolic or mitochondrial disorder (10 out of 17, 58.8%), followed by autosomal recessive sarcomeric CM (three; 17.6%), severe neonatal Rasopathy (three; 17.6%), and one (5.9%) ultra-rare syndromic disorder.

### 3.3. Impact on Clinical Management, Diagnostic Yield and Genetic Diagnosis in Urgent vs. Non-Urgent Cases

The achievement of the genetic diagnosis contributed to clinical management and decision-making in all 17 cases with a positive trio-WES result provided in urgency (Table 3). In six patients (35.3%), it allowed starting a specific medical treatment such as enzymatic replacement therapy (ERT) in Pompe disease, Nitisinone with appropriate diet for tyrosinemia, hormone replacement for pituitary hormone deficiency, and dietary management in trifunctional protein deficiency. In seven (41.2%) of them, the underlying condition did not contraindicate HTX, and, to date, four patients were transplanted with good results (Figure 2 and Figure 3). In four patients (23.5%), the molecular diagnosis of a disease with a poor prognosis associated with the critical conditions of patients led to palliative care. In addition, in four out of seven cases (57.1%) which remained undiagnosed, the negative result of trio-WES analysis was considered an element of non-contraindication to an eventual HTX, since it allowed for the general exclusion of several conditions for which HTX would be unsuccessful. Moreover, in nine patients, WES results provided an indication for the specific monitoring of extra-cardiac manifestations.

To date, 14 out of the 24 critical patients (58.3%) deceased: seven (50%) were affected by an inborn error of metabolism, two (14.3%) by a severe form of Rasopathy, one (7.1%) by a sarcomeric CM due to TNNI3 biallelic variants, and four (28.6%; two suspected syndromic CMs and two suspected inborn error of metabolism) remained undiagnosed. On the contrary, nine (37.5%) are alive and in stable clinical conditions (average follow-up of 24 months), and one was lost at follow-up after HTX. The patient with tyrosinemia underwent liver transplantation for a failure of specific medical treatment.

In 16 (88.9%) out of the 18 clinically stable patients in which trio-WES was performed with standard delivery time (average time of 225 days, range 46–452), a negative test (four cases) or the achievement of a conclusive diagnosis (12 patients) still had an impact on long term clinical management, due to excluded contraindications to eventual HTX. To date, 13 (72.2%) of these 18 patients are alive and clinically stable, two (11.1%) were transplanted and still alive, one deceased, and two were lost at follow-up.

In general, the outcome of patients was heavily influenced by the presence of extracardiac features and admission in ICU. 

Totally, in 37 patients (88.1%), the trio-WES results contributed to clinical management decision-making, including HTX, palliative care, or medical treatment.

**Figure 5:**
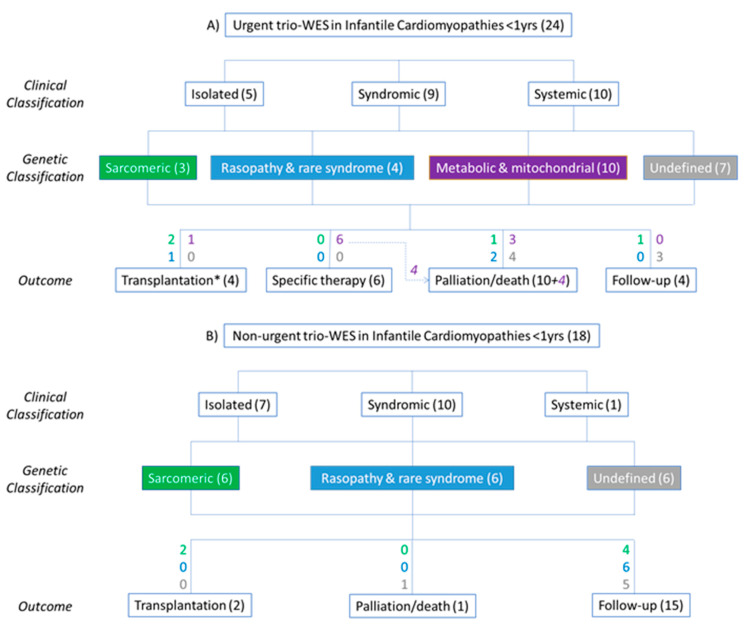
Flowchart describing the main results of trio-WES analysis in infantile cardiomyopathies, performed in urgent (**A**) and non-urgent (**B**) settings; * 1 patient underwent liver transplantation (tyrosinemia).

The diagnostic yield was comparable between urgent (70.8%) and non-urgent setting (66.7%), although the distribution of genetic causes was different. Indeed, isolated forms due to single, often de novo, sarcomere gene mutations were more frequent in stable patients, while in ICU, complex metabolic or mitochondrial disorders were the most represented. All these results are summarized in Figure 5.

## 4. Discussion

Here, we report a broad and heterogeneous spectrum of genetic disorders leading to infantile CMs, showing the great utility of WES for rapid diagnosis and consequently prompt clinical management. 

The clinical utility of WES has been demonstrated in both “urgent” and “non-urgent” cohorts, especially regarding the choice to include the patients in heart transplantation programs. However, the real impact of this comprehensive analysis is much more evident in critically ill infants. Indeed, a timely genetic diagnosis in these patients helps to immediately start a targeted therapy or diet, which, if delayed, could determine the irreversibility of the condition. For those conditions associated with poor prognosis, it has been possible to redirect treatment towards palliative support by reducing invasiveness. Furthermore, it should not be forgotten that in a patient in critical condition with a high probability of a fatal outcome, the search for an etiological diagnosis is mandatory because it can have a significant impact on family counseling.

The broad clinical and genetic heterogeneity of infantile cardiomyopathies, the rarity of their underlying conditions, and the problematic clinical evaluation of critical patients often challenge the diagnosis solely based on clinical grounds [27,28]. Indeed, numerous clinical, instrumental, biochemical, metabolic, and invasive tests, as well as cascade genetic tests, are often performed, with a considerable waste of time and resources and poor effectiveness for the immediate clinical management of patients. In our experience, trio-WES analysis in infants with CM is clinically helpful in timely reaching a definitive diagnosis and contributing to clinical decision-making.

In our study, we observed a total diagnostic yield of 69%. Among the 29 diagnosed patients, only nine had isolated sarcomeric CM, three of which had homozygous truncated variants leading to the complete absence of the troponin I protein in heart muscle, while one was compound heterozygous of variants in two different sarcomeric genes. Of the remaining five cases, two had de novo variants, while only three infants had inherited variants of sarcomeric genes from an affected parent. Recessive sarcomeric forms are more frequent in infantile CM than in adult patients and are associated with a poor prognosis demanding prompt transplantation. Moreover, excluding the TNNI3 recessive forms, no patients with sarcomeric gene variants were in critical clinical condition.

Our data strengthen the evidence that the genetic bases of infantile CMs are quite different from those of adults [29]. Specifically, we identified inborn errors of metabolism and mitochondrial disorders in 10 patients, all admitted in ICU, and syndromic CMs in another 10 cases, showing that sarcomeric CMs represent only a small proportion of infantile CMs. These findings suggest that traditional testing with gene panel sequencing usually performed in adult CMs patients is not suitable for testing infantile CMs since they do not provide a complete, unbiased, and updated result [12].

Furthermore, in three cases, the molecular diagnosis would not have been reached if a traditional testing strategy or commercial genetic panel had been performed due to the rarity of the conditions, the type of alteration, or the recent identification of the causative gene (*NONO*, *HCCS*, and duplication of *ATAD3*). 

Pediatric CMs, similarly to all rare pediatric diseases, have greatly benefited from identifying novel disease genes thanks to the massive use of WES. Indeed, new genes associated with complex forms of pediatric CM are constantly reported in the literature, making WES the most complete and updated test that allows reaching a timely and clinically helpful diagnosis [30].

Limitations of our study include a relatively small sample size and a potential bias in patient enrollment due to the multicenter design of the study. Indeed, our results should not be considered representative of the actual distribution of etiological bases and clinical presentations of infantile CM, but they are probably biased towards more severe forms.

Due to the rarity of CMs < 1 year, our patient cohort was collected and analyzed over eight years, during which there were technological improvements. This could have impacted non-urgent cases, as about half were enrolled before 2016, while analyses of all urgent cases have been performed after 2016. However, this aspect does not seem to have impacted the diagnostic yields, which are very similar between the two time periods.

## 5. Conclusions

Trio-WES analysis significantly impacts the diagnosis and contributes to the clinical management of infantile cardiomyopathies. Moreover, WES should be considered an essential part of clinical decision-making and should be performed promptly to maximize its clinical utility.

## Figures and Tables

**Table 1:** Patients’ population characteristics.

	All	Urgent	Non Urgent
**Patients *n***	42	24	18
**Gender**			
Male *n* (%)	26 (61.9%)	12 (50%)	14 (77.8%)
Female *n* (%)	16 (38.1%)	12 (50%)	4 (22.2%)
**Mean age (median) in months**	4.0 (3.0)	4.3 (3.0)	3.7 (3.0)
**CM type**			
HCM *n* (%)	30 (71.4%)	19 (79.2%)	11 (61.1%)
DCM *n* (%)	12 (28.6%)	5 (20.8%)	7 (38.9%)
**CM classification**			
Isolated *n* (%)	12 (28.6%)	5 (20.8%)	7 (38.8%)
Complex *n* (%)	30 (71.4%)	19 (79.2%)	11 (61.2%)
**Positive family history *n* (%)**	5 (11.9%)	3 (12.5%)	2 (11.1%)
**Prenatal diagnosis of CM *n* (%)**	9 (21.4%)	5 (20.8%)	4 (22.2%)

**Table 2:** Clinical presentation and list of identified mutations in our pediatric CM patients.

ID#	Urgent	Age (Months)	Sex	Type of CM *	Disease	Gene	Genomic Position (hg19)	cDNA	Protein	Zygosity	Inheritance	ACMG Classification
2	no	7	M	iDCM	Cardiomyopathy, dilated	*ACTC1*	Chr15:g.35085595T>C	NM_005159.4:c.305A>G	p.(Glu102Gly)	hetero	father	Likely pathogenic
3	no	3	F	iDCM	Cardiomyopathy, dilated	*TNNT2*	Chr1:g.201331116C>T	NM_001001430.2:c.614G>A	p.(Arg205Gln)	hetero	de novo	Pathogenic
5	yes	1	M	iHCM	Noonan syndrome	*PTPN11*	Chr12:g.112888202C>T	NM_002834.4:c.218C>T	p.(Thr73Ile)	hetero	de novo	Pathogenic
6	no	1	M	sHCM	Noonan syndrome	*RAF1*	Chr3:g.12645682_12645684del	NM_001354689.1:c.785_787del	p.(Asn262_ Val263delinsIle)	hetero	de novo	Pathogenic
7	yes	1	M	sHCM	Glycogen storage disease of heart, lethal congenital	*PRKAG2*	Chr7:g.151257696C>T	NM_016203.4:c.1592G>A	p.(Arg531Gln)	hetero	de novo	Pathogenic
8	yes	12	F	iDCM	Cardiomyopathy, dilated	*TNNI3*	Chr19:g.55668662A>T	NM_000363.4:c.24+2T>A	skip of exon 2	homo	both	Likely pathogenic
12	yes	4	F	mHCM	Trifunctional protein deficiency	*HADHB*	Chr2:g.26502860A>G	NM_000183.3:c.812-2A>G	skip of exon 10	homo	both	Likely pathogenic
13	yes	6	F	iHCM	Glycogen storage disease II	*GAA*	Chr17:g.78078910del	NM_000152.5:c.525del	p.(Glu176fs*45)	hetero	father	Pathogenic
							Chr17:g.78090909G>A	NM_000152.5:c.2331+1G>A	skip of exon 16	hetero	mother	Pathogenic
14	yes	3	F	sHCM	Linear skin defects with multiple congenital anomalies	*HCCS*	ChrX:g.11139015G>A	NM_005333.4:c.522-12G>A	p.(Ala174fs*2)	hetero	de novo	Likely pathogenic
17	no	1	F	sHCM	Noonan syndrome	*PTPN11*	Chr12:g.112926270C>T	NM_002834.4:c.1403C>T	p.(Thr468Met)	hetero	de novo	Pathogenic
18	no	0	M	sHCM	Noonan syndrome	*RAF1*	Chr3:g.12645699G>A	NM_001354689.1:c.770C>T	p.(Ser257Leu)	hetero	de novo	Pathogenic
19	yes	3	M	mHCM	Tyrosinemia, type I	*FAH*	Chr15:g.80450512G>T	NM_000137.3:c.192G>T	p.(Gln64His)	homo	both	Pathogenic
20	yes	10	F	mHCM	Glycogen storage disease II	*GAA*	Chr17:g.78078910del	NM_000152.5:c.525del	p.(Glu176fs*45)	hetero	father	Pathogenic
							Chr17:g.78079671C>T	NM_000152.5:c.670C>T	p.(Arg224Trp)	hetero	mother	Pathogenic
21	yes	1	F	mHCM	Trifunctional protein deficiency	*HADHB*	Chr2:g.26505741del	NM_000183.3:c.962del	p.(Met321fs*17)	hetero	mother	Likely pathogenic
							Chr2:g.26502860A>G	NM_000183.3:c.812-2A>G	skip of exon 10	hetero	father	Likely pathogenic
22	yes	9	M	mDCM	Barth syndrome	*TAZ*	ChrX:g.153640263_153640266del	NM_000116.3:c.83_86del	p.(Val28Alafs*11)	hemi	mother	Likely pathogenic
24	yes	11	M	mHCM	Mucopolysaccharidosis-plus syndrome	*VPS33A*	Chr12:g.122717464G>A	NM_022916.6:c.1492C>T	p.(Arg498Trp)	homo	both	Pathogenic
26	yes	3	M	mHCM	Pituitary hormone deficiency	*POU1F1*	Chr3:g.87310439G>A	NM_000306.3:c.649C>T	p.(Arg217*)	homo	both	Likely pathogenic
27	yes	0	M	sHCM	Noonan syndrome	*PTPN11*	Chr12:g.112926908C>G	NM_002834.4:c.1528C>G	p.(Gln510Glu)	hetero	de novo	Pathogenic
28	yes	0	M	sHCM	Costello syndrome	*HRAS*	Chr11:g.534288C>T	NM_005343.4:c.35G>A	p.(Gly12Asp)	hetero	de novo	Pathogenic
30	yes	1	F	mHCM	Chromosome 1p36.33 duplication syndrome	*ATAD3A*	Chr1:1392270_1460317dup	Fusion gene ATAD3A-ATAD3C		hetero	de novo	Pathogenic
32	no	0	M	iHCM	Cardiomyopathy, hypertrophic	*MYL2*	Chr12:111356937C>T	NM_000432.3:c.64G>A	p.(Glu22Lys)	hetero	mother	Pathogenic
					Cardiomyopathy, hypertrophic	*MYH7*	Chr14:g.23887522C>T	NM_000257.3:c.4066G>A	p.(Glu1356Lys)	hetero	father	Pathogenic
33	no	5	M	sHCM	Noonan syndrome	*PTPN11*	Chr12:g.112910827A>G	NM_002834.4:c.836A>G	p.(Tyr279Cys)	hetero	de novo	Pathogenic
35	no	2	M	iDCM	Cardiomyopathy, dilated	*MYH7*	Chr14:g.23886789C>T	NM_000257.3:c.4276G>A	p.(Glu1426Lys)	hetero	mother	Likely pathogenic
36	no	12	M	iHCM	Cardiomyopathy, hypertrophic	*MYH7*	Chr14:g.23886717C>T	NM_000257.3:c.4348G>A	p.(Asp1450Asn)	hetero	de novo	Likely pathogenic
37	no	7	M	iDCM	Cardiomyopathy, dilated	*MYH7*	Chr14:g.23887513G>A	NM_000257.3:c.4075C>T	p.(Arg1359Cys)	hetero	father	Likely pathogenic
38	no	8	F	sHCM	Noonan syndrome	*PTPN11*	Chr12:g.112910827A>G	NM_002834.4:c.836A>G	p.(Tyr279Cys)	hetero	de novo	Pathogenic
40	no	3	M	sDCM	Intellectual developmental disorder, X-linked syndromic	*NONO*	ChrX:g.70514185C>T	NM_001145408.1:c.457C>T	p.(Arg153*)	hemi	mother	Pathogenic
41 ^§^	yes	9	F	iDCM	Cardiomyopathy, dilated	*TNNI3*	Chr19:g.55667648del	NM_000363.4:c.204del	p.(Arg69fs*8)	homo	both	Likely pathogenic
42 ^§^	yes	10	F	iDCM	Cardiomyopathy, dilated	*TNNI3*	Chr19:g.55667648del	NM_000363.4:c.204del	p.(Arg69fs*8)	homo	both	Likely pathogenic

*: type of CM, i: isolated, m: metabolic, s: syndromic. ^§^ these are 2 unrelated patients belonging to different ethnic groups (patient 41 is from Morocco and patient 42 is from north Italy).

**Table 3:** Contribution to clinical management of urgent trio-WES results (number of infants).

Treatment	*n*.
Non-contraindication to heart transplantation	11 *
ERT/diet/specific therapy	6
Palliative care	4
Specific follow-up for extra-cardiac manifestations	9

* Liver transplantation in 1 case (tyrosinemia).
